# GANT61/BI-847325 combination: a new hope in lung cancer treatment

**DOI:** 10.1007/s12032-022-01738-4

**Published:** 2022-07-14

**Authors:** Abdel Halim M. El-Kishky, Nermine Moussa, Maged W. Helmy, Medhat Haroun

**Affiliations:** 1 Department of Biotechnology, Institute of Graduate Studies and Research, Alexandria University, Alexandria, Egypt; 2 Department of Pharmacology and Toxicology, Faculty of Pharmacy, Damanhur University, Damanhur, Egypt

**Keywords:** Lung cancer, MAPK, Aurora Kinase, Hedgehog, GANT61, BI-847325

## Abstract

Despite the huge efforts employed to implement novel chemotherapeutic paradigms for lung cancer, the disease still remains a major concern worldwide. Targeting molecular pathways as Hedgehog (Hh) and Mitogen-activated protein kinase (MAPK) represent a new hope in lung cancer treatment. This work was undertaken to evaluate the antitumor effects of GANT61 (5 μM), BI-847325(30 μM), and GANT61 (5 μM)/BI-847325(30 μM) combination on A549 adenocarcinoma lung cancer cell line. The growth inhibition 50 (GI50) for both drugs was performed using MTT. The protein levels of Caspase-3, Bcl-2-associated X protein (Bax), Myeloid cell leukemia sequence 1 (MCL-1), cyclin D1, vascular endothelial growth factor (VEGF), extracellular signal-regulated kinases (ERK), p-Akt, and phosphohistone H3 (pHH3) were measured using ELISA. Glioma-associated oncogene homolog 1(*Gli1*) gene expression was assessed by quantitative real-time PCR. The GI50 for GANT61 and BI-8473255 were 5 µM and 30 µM, respectively. Caspase-3 and Bax protein levels were significantly elevated while MCL-1, cyclin D1, VEGF, ERK 1/2, p-Akt, and pHH3 levels were significantly reduced by both drugs and their combination relative to the control group. *Gli1* gene expression was down-regulated in all groups relative to the control group. GANT61, BI-847325 and their combination inhibited proliferation and angiogenesis but activated the apoptotic pathway. Both drugs conferred a profound negative impact on the crosstalk between each of Hh and MAPK pathways and Phosphoinositide *3 *-kinases (PI3K)/Akt/Mammalian target of Rapamycin (mTOR). To the best of our knowledge, the antitumor effects of BI-847325/GANT61 combination have not been tested before. Further in-vitro and in-vivo studies are warranted to support the findings.

## Introduction

According to Global Burden of Cancer Study (GLOBOCAN) 2018, lung cancer is the most frequent cancer in both sexes in the estimated new cases with an overall ratio of mortality to incidence of 0.84 [1]. The 5-year relative survival rate is 16.3% which is very low compared to other cancers [3]. The high mortality due to lung cancer is mainly ascribed to its late diagnosis which decreases the potential for treatment success [2].

Genetic deregulation is shown in all types of lung cancer and involves different sets of mutated genes and deregulated cellular signaling pathways. These alterations include receptor tyrosine kinase signal transduction pathway, PI3K pathway, and *TP53*, the most mutated gene in all types of lung cancer. These altered pathways are involved in the initiation, progression, and even recurrence of lung cancer. Consequently, targeting signaling pathways with novel drugs may be a hope for lung cancer treatment [4].

Hedgehog (Hh) pathway is responsible for cell differentiation and embryonic development [5]. It has a role in stem cell renewal and organ homeostasis. *Gli1* is over-activated in various cancers including lung cancer. Gli1, downstream of Hh pathway, acts as transcriptional factor for several genes involved in cell proliferation and survival [6]. GANT61 is an inhibitor for Gli1-induced transcriptional inhibition of Hh pathway. GANT61 targets many of the classical hallmarks of cancer. GANT61decreases cell viability and inhibits proliferation. GANT61 inhibits epithelial-mesenchymal transition, stimulates autophagy of cancer cells, stimulates the immune system, and induces apoptosis [5, 7].

Mitogen-activated protein kinase (MAPK) pathway regulates cell proliferation, survival, differentiation, angiogenesis, and migration. It is a tyrosine kinase-dependent pathway that transduces the signal sequentially via rat sarcoma viral oncogene (RAS), rapidly accelerated fibrosarcoma, and ERK. Mutations in the MAPK pathway have a major role in lung cancer [8]. Aurora kinase acts as a mitotic checkpoint and is considered a promising target for cancer treatment. Aurora kinase inhibitors cause chromosomal instability and because of cell division failure, cells undergo apoptosis or arrest in a pseudo G1 state [9].

BI-847325 selectively binds to and inhibits the activity of MAPK, which prevents the activation of MAPK downstream effector proteins and inhibits proliferation and survival of cancer cells. Moreover, BI-847325 binds to and inhibits the activity of the pan Aurora kinases which inhibit both cell division and proliferation. So, BI-847325 can inhibit and retard cell survival and tumor growth [10].

Accordingly, the objective was to assess the potential antitumor effects of GANT61, BI-847325 as well as their combination in A549 lung adenocarcinoma cell line.

## Materials and methods

### Drugs under study

BI-847325 and GANT61 (Selleck Chemicals, USA) were prepared as 10 mM stock solutions using dimethyl sulphoxide (DMSO).

### Cell line

A549 is an epithelial lung carcinoma cell line obtained from the American Type Culture Collection (ATCC® CCL185™). The A549 cell line was first developed in 1972 through the removal and culturing of cancerous lung tissue in the explanted tumor of 58-year-old Caucasian male.

### Cell thawing

Aliquots of complete growth medium (90% Dulbecco's Modified Eagle's Medium, 10% v/v fetal bovine serum) were warmed in a water bath at 37 °C. The cryovials were quickly thawed by gentle agitation in 37 °C water bath. The content of the cryovials were then transferred to a 15 ml falcon tubes containing 9 ml of pre-warmed complete medium. Cells were gently resuspended then were centrifuged at 12,000 rpm (radius of the rotor = 3.5 cm) (Nahita Minivit Centrifuge, Model 2716) for 10 min at 4 °C. The supernatant was removed by aspiration and cells were resuspended in 5 ml of complete medium.

### Cell cultures

A549 cells were maintained as a monolayer culture in T-25 flasks (Greiner Bio-One, Germany) at 37 °C and 5% CO_2_ in Dulbecco's Modified Eagle's Medium (Lonza Verviers SPRL, Belgium) supplemented with 10% (v/v) fetal bovine serum (PAA, Brazil). Penicillin/Streptomycin (Lonza Verviers SPRL, Belgium) were used at the concentrations of 100 units/ml and 100 µg/ml, respectively.

### Subculturing of cells

A549 cells were passaged when they were 80% confluent, about every third day. Media was removed by aspiration and 5 ml of phosphate buffered saline pH 7.2 (Lonza Verviers SPRL, Belgium) were added to wash adherent cells. To detach the adherent cells, 1 ml of 2.5% (w/v) Trypsin (Lonza Verviers SPRL, Belgium) was added to the T-25 flasks and cells were incubated for 5 min at 37 °C. Then, cells were detached and observed under the inverted microscope (Micro master inverted digital microscope, Thermo Fisher Scientific Inc., USA) every 2–3 min.

### Cell counting

To determine an inoculum with the appropriate cell concentration for seeding, cells were counted using the hemocytometer. Briefly, cell suspension (10 µl) was mixed with equal volume of trypan blue and loaded in both chambers. Unstained cells (viable cells) were counted under an inverted microscope at 10 × magnification. The total number of cells can be calculated as follows:

$$\begin{gathered} {\text{Cells}}/{\text{suspension}}\; = \;{\text{Total}}\;{\text{cells}}\;{\text{counted}}\;{\text{in}}\;{\text{the}}\;{\text{hemocytometer}}\;{\text{sets}}\;{\text{of}}\;{\text{squares}}/4{\text{ }} \times 10^{4} \hfill \\ \times \;{\text{dilution}}\;{\text{factor}}\; \times \;{\text{volume}}\;{\text{of}}\;{\text{cell}}\;{\text{suspension}}. \hfill \\ \end{gathered}$$

Cells/suspension = Total cells counted in the hemocytometer sets of squares/4 × 10^4^ × dilution factor × volume of cell suspension.

### Growth inhibition (GI50) assay

Inhibition of cell growth in response to GANT61 and BI-847325 was examined by Microculture Tetrazolium Test (MTT) [11]. The MTT cell viability assay measures the cell proliferation where the yellow Tetrazolium is reduced by metabolically active cells, yielding purple Formazan crystals which has a λ max at 540 nm and is directly proportional to the number of viable cells. Briefly, cells were plated at approximately 4000 cells/well in 96 well plates and incubated for 24 h at 37°c. After aspiration, the treatment medium (100 µl) was added. Different concentrations of GANT61 (0.625, 1.25, 2.5, 5, 10, 20 μM) and BI-847325 (3.75, 7.5, 15, 30, 60, 120 μM) were used. After addition of the tested drugs, the plate was incubated for 72 h. GI50 determination for GANT61 and BI-847325 was done using the Compusyn 3.0.1 software.

### Determination of the combination index

The combination index (CI) was assessed as described earlier [12] to determine whether there is synergism, antagonism, or additive effect between GANT61 and BI-847325, where CI lower than 1 indicates synergism, =1 indicates additive effect and greater than 1 indicates antagonism.

### Treatment of A549 cells with the selected drugs

Drugs were added on day 1 according to the following design: (a) Control A549 cells: treated with 1% DMSO in complete growth medium as vehicle; (b) GANT61 treated A549 cells: GANT61 was dissolved in 1% DMSO and diluted with complete culture medium to a final concentration of 5 µM; (c) BI-847325 treated A549 cells: BI-847325 was dissolved in 1% DMSO and diluted with complete culture medium to a final concentration of 1 µM; (d) GANT61/BI-847325 treated A549 cells: GANT61 and BI-847325 were dissolved in 1% DMSO and diluted with complete culture medium to final concentrations of 5 µM and 1 µM, respectively. On day 4, cells were collected and pellets were stored at –80 °C for the determination of the different parameters.

### Total protein content determination

Total protein content was assayed as previously described [13]. Bovine serum albumin (BSA) was used as the standard and was dissolved in the cell lysis solution at the concentrations of 0.13, 0.25, 0.5, 1, 2, 3, and 4 mg/ml. The cell lysates were diluted 10 times by the cell lysis buffer. Duplicates of 10 ml aliquots of the standard solutions and diluted lysates were pipetted into disposable glass tubes followed by the addition of 2.5 ml of the Bradford reagent. All tubes were vortexed and were then left for 2 min at room temperature and the absorbance was measured using a spectrophotometer against blank within one hour at 595 nm. A standard curve was graphed by plotting the concentrations of the standard on the X-axis and the absorbance on the Y-axis. The protein concentration of the diluted lysate was then estimated from the standard curve by extrapolation.

### Preparation of cell lysate for ELISA

Cell lysis solution (1 ml) and 1 × Sigma FAST™ protease inhibitor tablet solution (1 ml) (Sigma-Aldrich, Germany) (CAT#: S8820) were added to A549 cell pellets and then kept for 2.5 h on ice with frequent vortexing. Cell lysates were centrifuged at 12,000 rpm for 5 min at 4ºC and supernatants were obtained and stored at − 20 °C.

### Biochemical analyses using ELISA technique

The protein levels of cyclin D1, Bax, VEGF, p-Akt, ERK, pHH3, and MCL-1 were determined using the following ELISA kits (Lifespan Biosciences, Inc., USA) (Cat#: LS- F4095); (Lifespan Biosciences, Inc., USA) (Cat#: LS-F5064); (Cusabio, USA) (Cat#: CSB-e11718h); (Raybiotech, USA) (Cat#: PEL-Akt-S473-T); (Raybiotech, USA) (Cat#: CBEL-ERK-1); (Invitrogen, USA) (Cat#: KHO0671); (Elabscience Biotechnology Co., Ltd, USA) (Cat#: E-EL-H1734), respectively according to the manufacturer^’^s instructions.

Briefly, 100 μl of samples or standards were added, then, incubation was carried out for 2 h at room temperature. A working solution (100 μl) was added; wells were covered and agitated gently to ensure thorough mixing. After that, incubation was carried out for 1 h at 37 °C followed by aspiration and washing for 3 times by adding approximately 350 μl of 1× wash buffer. After that, detection reagent B working solution (100 μl) was added followed by incubation for 30 min at 37 °C, aspiration and washing for 5 times as outlined previously.

TMB substrate solution (90 μl) was added to each well followed by incubation for 10–20 min at 37 °C. Then, stop solution (50 μl) was added to each well. The optical density (OD value) was determined using a microplate reader set to 450 nm. A standard curve was constructed by plotting the concentrations of the standard on the X-axis and the absorbance on the Y-axis.

### Determination of active Caspase-3 in A549 cell lysates

Human Caspase-3 assay kit (Sigma Aldrich, USA) (Product Code CASP-3-C) was used. It is based on the hydrolysis of the peptide substrate acetyl-Asp-Glu-Val-Asp p-nitroanilide (Ac-DEVD-pNA) by Caspase-3, resulting in the release of the p-nitroaniline (pNA) moiety. The concentration of the pNA released from the substrate was calculated from the absorbance values at 405 nm.

Briefly, cell lysate or Caspase-3 positive control (10 μl) were placed in the appropriate tubes and 1× assay buffer was added as indicated in the manufacturer ‘s instructions. After that, Caspase-3 inhibitor (10 μl) was added and the reaction was started by adding 10 μl of Caspase-3 substrate. The tubes were then covered and incubated at 37 °C for 1.5–2 h and absorbance was read at 405 nm. Caspase-3 activity was calculated in mmol of pNA released per minute per ml of cell lysate or positive control based on the formula:

$${\text{Activity}}\; = \;\frac{{{\text{OD}}\; \times \;{\text{d}}}}{{{\text{T}}\; \times \;{\text{V}}\; \times \;\varepsilon^{{{\text{Mm}}}} }}$$

where Molar absorptivity (Mm) = 10.5, V: Volume of sample in ml, D: Dilution factor T: Reaction time in minutes.

## *Gli1* gene expression analysis using qRT-PCR

Total RNA was extracted using Easy-spin™ total RNA extraction kit (Intron Biotechnology, South Korea) (Cat #: 17,221) followed by the determination of RNA quantity and purity using NanoDrop 2000 spectrophotometer (Thermo Fischer Scientific, USA). Quantitative RT-PCR was performed using the SensiFast™ SYBR® No-ROX one-step kit (Bioline Co., USA) (Cat #: BIO-72001). The sequences of the forward and reverse primers for *Gli1*gene were F: 5'- TTCCTACCAGAGTCCCAAGT-3' and R: 5'-CCCTATGTGAAGCCCTATTT-3', whereas those for the housekeeping gene (*β-actin)* were F: 5'-CTGGAACGGTGAAGGTGACA-3' and R: 5'-AAGGGACTTCCTGTAACAATGCA-3' [14, 15]. To confirm the expected unique amplification of *Gli1* and *β-actin* genes, the sequences of the primers were blasted against NCBI/Primer Blast. The analyses were performed as triplicates. The relative expression level of *Gli1* gene against *β-actin* as a housekeeping gene depended on ∆∆CT method.

### Statistical analysis of the data

Data was expressed as means ± standard error of the mean. Multiple comparisons were analyzed using one-way analysis of variance (ANOVA) followed by the post hoc test and *P* < 0.05 was set as the level of significance. Statistical tests were carried out using Graph Pad Prism**®** software package version 6 (GraphPad Software Inc., USA).

## Results

### Determination of the GI50 of GANT61 and BI-847325 in A549 cells

**Fig. 1:**
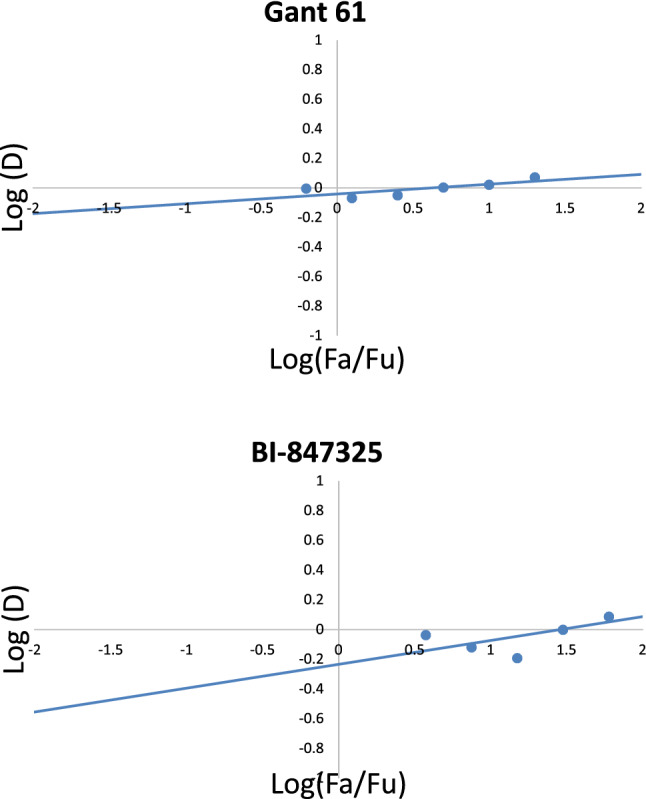
**A** Median-effect curve for GANT61, **B** median-effect curve for BI-847325 f_a_ affected fraction f_u_ unaffected fraction

GANT61 and BI-847325 exhibited potent cytotoxic effects as demonstrated in (Fig. 1). Exposure of A549 cells to both drugs resulted in a dose-dependent inhibition of cell viability with GI50 values of 5 μM and 30 μM for GANT61 and BI-847325, respectively, using the previously mentioned tested concentrations.

### Determination of the combination index

Based on the MTT assay and the statistical analyses using Compusyn software, GANT61/BI-847325 combination showed a synergistic effect as evidenced from the combination index (CI = 0.94 ± 0.01 μM).

### Effect of GANT61 (5 μM), Bi-847325 (30 μM), and GANT61 (5 μM)/ Bi-847325 (30 μM) combination on pathways crosstalk markers (p-Akt protein, ERk1/2 protein, and *Gli1* gene expression) in A549 cells after 72 h of treatment

**Fig. 2:**
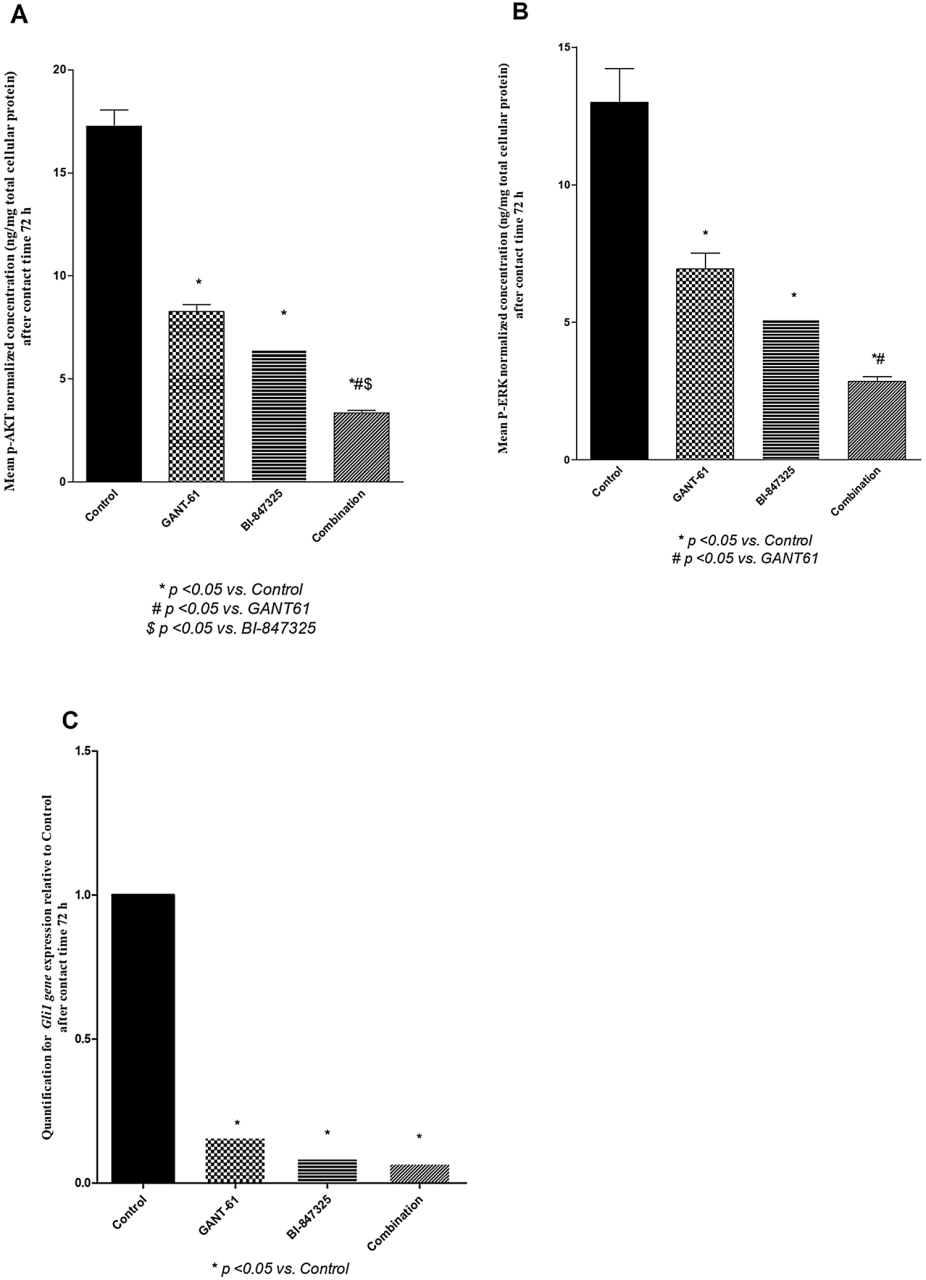
**A** Effect of GANT61 (5 μM) BI-847325 (30 μM) and their combination on p-Akt protein level (ng/mg total protein) in A549 cell lysates after contact time of 72 h, **B** Effect of GANT61 (5 μM) BI-847325 (30 μM) and their combination on ERK 1/2 protein level (ng/mg total protein) in A549 cell lysates after contact time of 72 h. **C** Effect of GANT61 (5 μM) BI-847325 (30 μM) and the combined treatment on *GLI1* gene expression level in A549 cell lysates after contact time of 72 h

Protein levels of p-Akt were reduced in all the treated groups relative to the control group (P < 0.0001) and the combination significantly inhibited p-Akt protein level compared with each drug alone (p < 0.05) as shown in (Fig. 2A). Likewise, Erk 1/2 protein was decreased in all the treatment groups relative to the control group (P < 0.0001) and the combination significantly inhibited Erk 1/2 when compared with GANT61treatment (p < 0.05) but not BI- 847,325 (p > 0.05) as illustrated in (Fig. 2B). Moreover, there was a significant inhibition of *Gli1*gene expression in all the treatment groups relative to the control group (P < 0.0001) as depicted in (Fig. 2C).

### Effect of GANT61 (5 μM), Bi-847325 (30 μM), and GANT61 (5 μM)/ Bi-847325 (30 μM) combination on cell death biomarkers (Caspase-3, BAX, and MCL-1), proliferation markers (cyclin D1 and pHH3) and angiogenic marker (VEGF) in A549 cells after 72 h of treatment

**Fig. 3:**
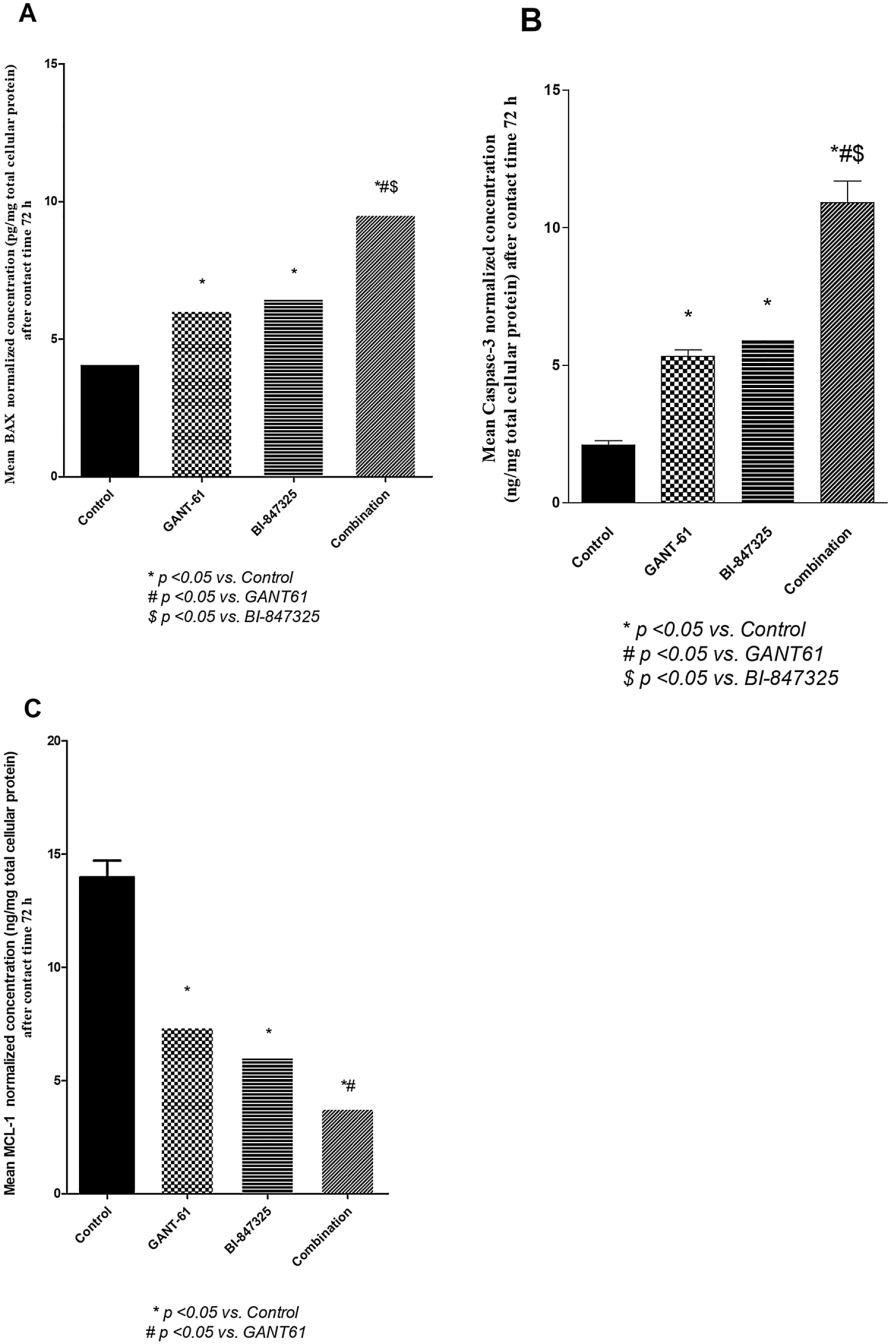
**A** Effect of GANT61 (5 μM) BI-847325 (30 μM) and their combination on BAX protein level (pg/mg total protein) in A549 cell lysates after contact time of 72 h. **B** Effect of GANT61 (5 μM) BI-847325 (30 μM) and their combination on active Caspase-3 protein level (ng/mg total protein) in A549 cell lysates after contact time of 72 h. **C** Effect of GANT61 (5 μM) BI-847325 (30 μM) and their combination on MCL-1 protein level (ng/mg total protein) in A549 cell lysates after contact time of 72 h

The lowest concentrations of BAX and active Caspase-3 were in the untreated A549 cells as compared to the other groups as depicted in (Fig. 3A, B). Noteworthy, the effect of the combination regimen overrides that of single drug treatments (P < 0.05) regarding BAX and active Caspase-3. Moreover, MCL-1 protein levels were significantly lower in all the treatment groups relative to the control group (P < 0.0004) and the combination significantly inhibited MCL-1 protein levels when compared with GANT61treatment (p < 0.05) but not BI- 847,325 (p > 0.05) as shown in (Fig. 3C).

**Fig. 4:**
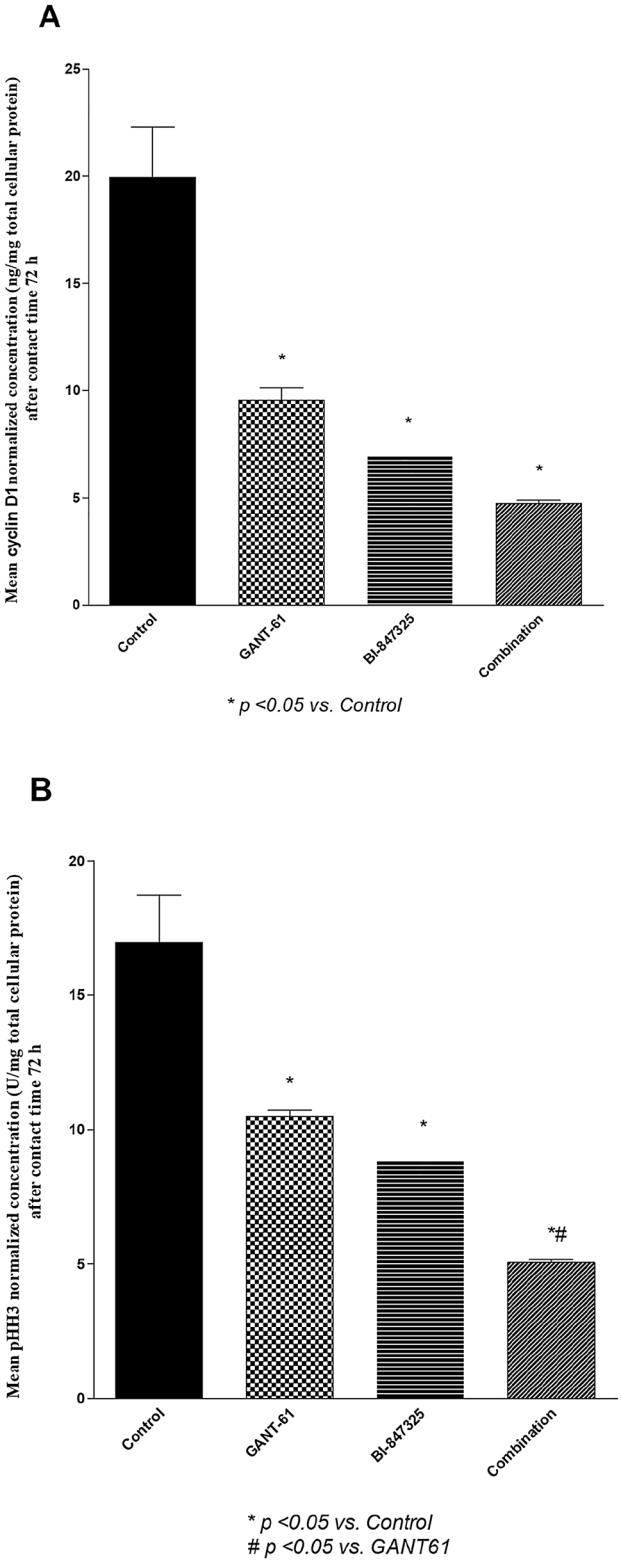
**A** Effect of GANT61 (5 μM) BI-847325 (30 μM) and their combination on cyclin D1 protein level (ng/mg total protein) in A549 cell lysates after contact time of 72 h. **B** Effect of GANT61 (5 μM) BI-847325 (30 μM) and their combination on pHH3 protein level (U/mg total protein) in A549 cell lysates after contact time of 72 h

**Fig. 5:**
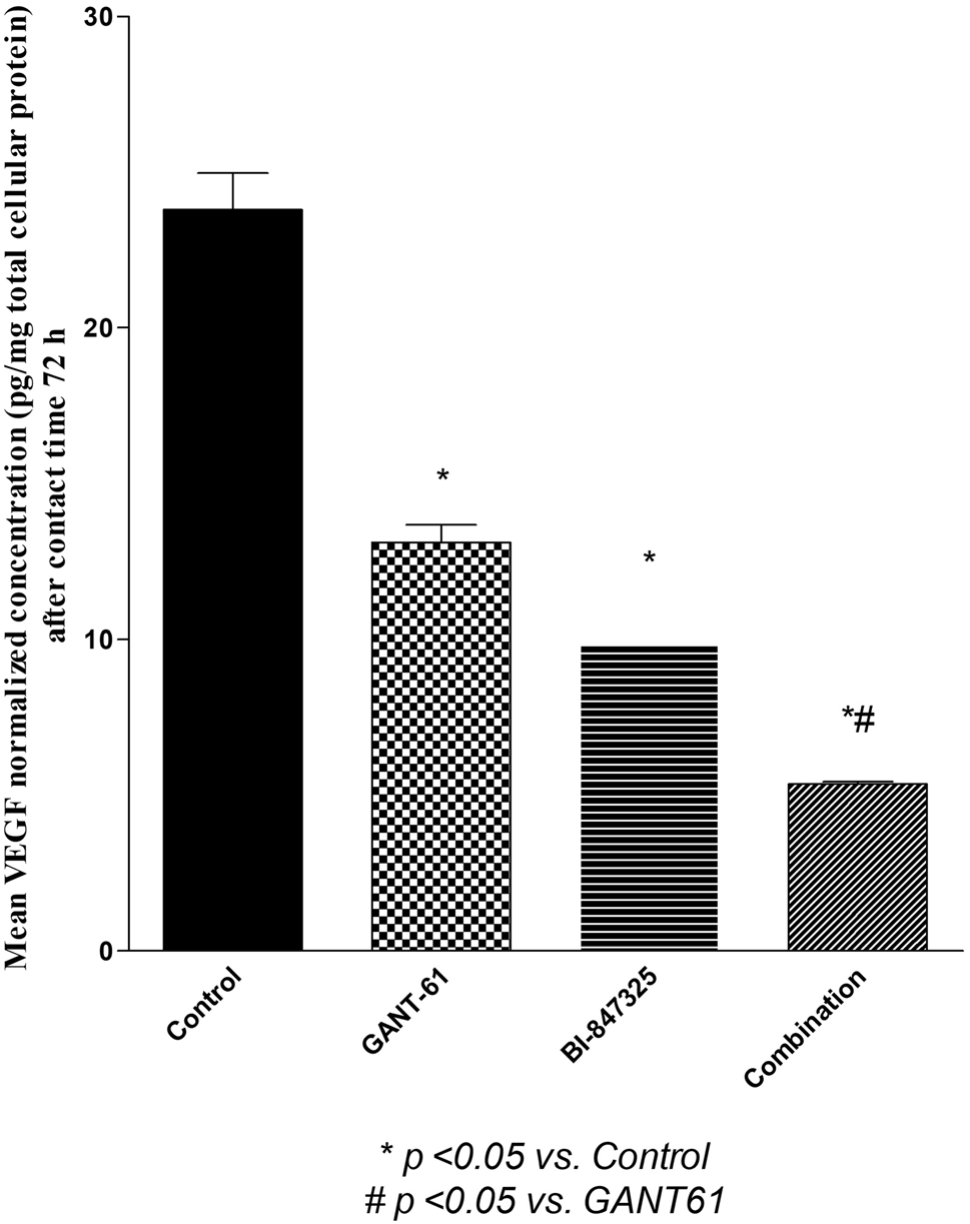
Effect of GANT61 (5 μM) BI-847325 (30 μM) and their combination on VEGF protein level (pg/ mg total protein) in A549 cell lysates after contact time of 72 h

Figure 4A showed that GANT61, BI-847325 and their combination significantly reduced cyclin D1 protein levels relative to the control group by 52%, 65%, and 76%, respectively (P < 0.0031). Figure 4B inferred that pHH3 protein levels were significantly lower in all the treatment groups relative to the control group (P < 0.0034). Likewise, GANT61, BI-847325, and their combination reduced VEGF levels by 45%, 59%, and 77.5% respectively (*P* < 0.0003) as shown in (Fig. 5). Notably, the combination significantly decreased pHH3 and VEGF levels when compared with GANT61treatment (p < 0.05) but not BI- 847,325 (p > 0.05).

## Discussion

Several lines of evidence supported critical roles for Hedgehog and MAPK signaling in lung cancer [16]. Accordingly, there was a growing interest in studying Hedgehog and MAPK pathways via their co-targeting by GANT61 (an inhibitor of downstream targets of Hh pathway) and BI-847325 (a dual MEK and Aurora kinase inhibitor). To the best of our knowledge, this study is the first to assess the possible antitumor effects of GANT61/BI-847325 combination in A549 adenocarcinoma lung cancer cell line. The synergistic effect of GANT61and BI-847325 was confirmed as indicated by the cell viability assay and calculated CI.

Pertaining to *Gli1* gene expression, both drugs and their combination blocked Hh pathway by down-regulating *Gli1* gene expression. The inhibitory effect of GANT61 was supported by a previous study which reported that Gli1 protein acts as a transcriptional factor for many genes including *Gli1* gene itself [17]. BI-847325 inhibited *Gli1* gene expression through the inactivation of MAPK/ERK/Gli1 non-canonical pathway. A previous study conducted on normal mouse embryo fibroblast cell line (NIH/3T3) reported the non-canonical activation of Gli and showed that the expression and transcriptional activity of Gli1 protein and the induction of Gli target genes was a response of the activated MAPK/ERK pathway [18]. Additionally, it was demonstrated that MAPK pathway increased the transcriptional factors and regulated Gli1 through Hh/Suppressor of Fused (SUFU)-independent pathway in gastric cancer cell lines [19].

Our results inferred that both drugs and their combination lowered p-Akt protein levels. Inhibition of p-Akt by GANT61 suggests that Gli1 is upstream of p-Akt. Concordant with the present findings, a previous study reported that over-expression of p-Akt is related to Gli1 protein over-expression in acute myeloid leukemia cells [20]. Furthermore, it was reported that Gli1 acts as a transcriptional factor to p-Akt as two Gli1 binding sites (BS1 and BS2) are present in the Akt1 promoter [21]. Inhibition of p-Akt protein by BI-847325 opposed the hypothesis that RAS/ERK and PI3K/Akt pathways negatively regulate each other’s activity as the activation of MAPK pathway blocks PI3K/Akt/mTOR pathway and vice versa [22]. p-Akt is activated by the inhibition of MAPK protein resulting in decreased phosphorylated epidermal growth factor receptor at the feedback site T669 which increases the activity of epidermal growth factor receptor and simultaneously activating the PI3K/Akt pathway [23]. BI-847325 is a dual MAPK/Aurora kinase inhibitor so the mechanism by which BI-847325 inhibits p-Akt protein is through Aurora kinase inhibition. This hypothesis is in line with a study that highlighted that Aurora kinase inhibitors decreased p-Akt protein level [24]. Furthermore, p-Akt is phosphorylated by Aurora kinase A in human bone osteosarcoma epithelial cells (U2OS) [25]. Notably, GANT61/BI-847325 combination has different mechanisms of action that led to the inhibition of p-Akt thus decreasing the potentiality of the cancer cells to develop resistance to such combination.

Our data revealed that both drugs and their combination inhibited p-ERK 1/2. The inhibition of p-ERK 1/2 by GANT61 is an indication for the inhibition of the crosstalk between Hh and MAPK pathways. There was an evidence for ERK upregulation by Hh signaling pathway in hepatocellular carcinoma [26]. Our results corroborate well with a previous study that inferred that Gli1 activated platelet-derived growth factor receptor (PDGFR α) leading to the activation of RAS/ERK pathway in basal cell carcinoma [27]. Therefore, inhibition of Gli1 by GANT61 inhibited ERK1/2 and decreased the proliferation of A549 cells. BI-847325 reduced the level of p-ERK 1/2 by the direct inhibition of MAPK protein which is upstream of ERK protein. ERK 1/2 is phosphorylated at the Thr and Tyr residues specifically by MAPK [28]. ERK 1/2 inhibition caused by the combination treatment is mainly attributed to the action of BI-847325 because ERK is downstream to MAPK protein [29].

Apoptosis deregulation plays a key role in cancer development and progression. This study assessed the effect of both drugs and their combination on the apoptotic pathway by determining the protein levels of BAX, MCL-1, and Caspase-3. Previous studies found a relationship between the expression of Bcl-2 family proteins and Hh pathway. The activation of Hh/Gli pathway increases the expression of the anti-apoptotic protein Bcl-2 and decreases the expression of the pro-apoptotic protein BAX through PI3K/Akt/Bcl-2 pathway [30, 31]. The activation of the caspase-dependent mitochondrial apoptotic pathway was observed in LX-2 human hepatic stellate cell line when treated with GANT61 [32]. GANT61 induces cell death through induction of FAS signaling by inhibiting PDGFR α which is a Gli target gene and a FAS regulator that leads to the increase in the protein levels of cleaved Caspase-3, cleaved poly ADP ribose polymerase, and death receptor 5 [7, 32]. MAPK pathway inhibits the apoptotic pathway through the inactivation of Bcl-2-associated death promoter (BAD) and this allows the activation of Bcl-2 and the resistance of cancer cells to apoptosis [29]. In parental melanoma cells, BI-847325 induced apoptosis by decreasing the expression of MCL-1 at the transcriptional and translational levels.

MCL-1 is an anti-apoptotic protein that belongs to the Bcl family and is responsible for cancer cell survival and resistance to treatment [10]. Aurora kinase inhibitors as BI-847325 contributed to the activation of the apoptotic machinery. The inhibition or deletion of Aurora kinase leads to the formation of polyploid cells which is followed by stress response such as DNA damage and stimulation of p53 leading to apoptosis [9]. Silencing of Aurora kinase can induce apoptosis in osteosarcoma cells [33]. The inhibition of Aurora kinase leads to the induction of apoptosis with the inhibition of Akt, and down-regulation of *Bcl-XL* expression in human tongue squamous cancer cell line [24]. Additionally, the statistically significant effect of the combination treatment on active Caspase-3 and BAX protein levels may suggest a potentiating role of GANT61 to BI-847325 to promote apoptosis. The effect due to the combination may be ascribed to the activation of both extrinsic and intrinsic caspase-dependent apoptotic pathways.

Both drugs and their combination have the ability to cause cell cycle arrest by decreasing the expression of cyclin D1 and pHH3. Inhibition of cyclin D1 by GANT61 may be due to the inhibition of Gli1 which acts as a transcriptional factor for *cyclin D1* gene [34]. It was suggested that BI-847325 inhibited cyclin D1 through blocking MAPK pathway which is responsible for cell growth and replication. This was supported by a previous study which showed that Mini-chromosome maintenance complex component 7 up-regulates cyclin D1 through modulating MAPK signaling in hepatocellular carcinoma [35].

It was reported that pHH3 is an accurate marker for cells within the mitotic phase of the cell cycle in many cancer types [36]. The inhibitory effect of GANT61 and BI-847325 on pHH3 may be ascribed to the inhibition of ERK1/2/ Mitogen- and stress-activated protein kinase axis [37, 38]. Supporting our results, a previous study found a significant decrease in pHH3 in malignant pleural mesothelioma cultures treated with Hh antagonist but the mechanism of inhibition is unknown [39]. Moreover, BI-847325 may decrease pHH3 protein level through the inhibition of Aurora kinase since Aurora kinase B was a well-known primary mitotic kinase responsible for histone H3 phosphorylation on Serine 10 and 28 [40]. The cell cycle arrest caused by the BI-847325/GANT61 combination may be attributed basically to BI-847325 as it has a direct inhibitory effect on MAPK protein which is the only effector of ERK1/2.

Both drugs and their combination decreased angiogenesis by inhibiting VEGF. GANT61 decreased VEGF protein level by blocking Gli1 which acts as a direct transcriptional factor to VEGF [34]. BI-847325 decreased VEGF through the inhibition of the MAPK pathway. Since constitutive activation of ERK1/2 leads to increased VEGF expression [41]. These results were previously confirmed where MAPK protein stimulates VEGF secretion through the activation of hypoxia-inducible factor 1α in ovarian and prostate cancers [42]. Furthermore, inhibition of VEGF by GANT61/BI-847325 combination highlighted that angiogenesis inhibition is mainly due to inhibition of MAPK pathway by BI-847325.

To conclude, the results inferred that both drugs and their combination are capable of inhibiting cancer cell growth, survival, and angiogenesis. Furthermore, both drugs and their combination decreased the potential resistance of A549 cells to treatments by inhibiting the cross-talk between MAPK, PI3K/Akt/mTOR, and Hh pathways. To the best of our knowledge, GANT61/BI-847325 combination has not been tested before both in vivo and in vitro*.* GANT61/BI-847325 combination seems to be promising in lung cancer treatment though further in vivo and in vitro studies are warranted to verify the present results.

## Data Availability

The data generated and analyzed during the current study are available from the corresponding author upon request.
